# Magnetic Bead Chain-Based Continuous-Flow DNA Extraction for Microfluidic PCR Detection of *Salmonella*

**DOI:** 10.3390/mi12040384

**Published:** 2021-04-01

**Authors:** Yuhe Wang, Wuzhen Qi, Lei Wang, Jianhan Lin, Yuanjie Liu

**Affiliations:** Key Laboratory of Agricultural Information Acquisition Technology, Ministry of Agriculture and Rural Affairs, China Agricultural University, Beijing 100083, China; yuhewang@cau.edu.cn (Y.W.); wuzhen.qi@cau.edu.cn (W.Q.); wanglei123@cau.edu.cn (L.W.); jianhan@cau.edu.cn (J.L.)

**Keywords:** magnetic bead chains, continuous-flow DNA extraction, microfluidic PCR, *Salmonella* detection

## Abstract

Nucleic acid extraction is crucial for PCR detection of pathogenic bacteria to ensure food safety. In this study, a new magnetic extraction method was developed using 3D printing and magnetic silica beads (MSBs) to extract the target DNA from a large volume of bacterial sample and combined with microfluidic PCR to determine the bacteria. After proteinase K was added into a bacterial sample to lyse the bacteria and release the DNA, it was continuous-flow injected into the serpentine channel of the extraction chip, where magnetic silica bead chains had been formed in advance using a homogeneous magnetic field generated by two concentric semicircle magnets to capture the MSBs. Then, the flowing DNA was captured by the MSB chains, washed with alcohol, dried with gas, and eluted with deionized water to obtain the purified and concentrated DNA. Finally, the extracted DNA templates were injected into a microfluidic PCR chip with lyophilized amplification reagents and determined using a commercial qPCR device. The experimental results showed that the DNA extraction efficiency was more than 90%, and the lower detection limit of *Salmonella* was 10^2^ CFU/mL. This new *Salmonella* detection method is promising to provide the rapid, sensitive, and simultaneous detection of multiple foodborne pathogens.

## 1. Introduction

Foodborne illness is an important public health issue, and an estimated 600 million people fall ill after eating contaminated foods every year, causing 420,000 deaths according to the statistics of World Health Organization [1]. *Salmonella* is one of the leading causes for foodborne diseases [2], and the United States Department of Agriculture (USDA) claimed that the annual cost due to the infection of *Salmonella* in the US was about 4.1 billion dollars [3]. Therefore, rapid screening of *Salmonella* is critical to prevent the spread of foodborne diseases.

To date, many rapid methods have been developed to detect foodborne bacteria. Among them, polymerase chain reaction (PCR) has received increasing attention and been widely applied for bacteria detection due to its high sensitivity, less time, and high throughput [4,5,6]. With the advance of PCR technology, different PCR methods have been developed for the rapid detection of foodborne pathogens, such as qPCR [7,8], multiplex PCR [9,10,11], and microfluidic PCR [12,13,14,15]. In recent years, microfluidic qPCR has become a new research hotspot because it can automatically perform quantitative nucleic acid analysis in a shorter time using less volume of reagents and sample [16,17,18]. Many efforts have been made on microfluidic qPCR to achieve rapid and sensitive detection of foodborne pathogens [19,20,21]. However, nucleic acid extraction, an important precondition for PCR detection of pathogenic bacteria, does not attract sufficient attention. At present, available DNA extraction methods mainly include filtration and centrifugation. However, they either need complex procedures or lack sufficient efficiency. Thus, new extraction methods for the rapid and efficient separation and concentration of nucleic acids from complex food samples are urgently needed. 

In the past decades, magnetic silica beads (MSBs) have been often reported to extract nucleic acids released from lysed bacterial cells at high salt condition through electrostatic adsorption [22,23,24], which is followed by magnetic separation of the MSBs with DNA, a thorough wash with ethanol, and elution in a small volume of deionized water to obtain purified and concentrated DNA templates [25,26]. So far, most MSB-based nucleic acid extraction methods are only suitable for the separation and concentration of DNA from a small volume; however, this cannot meet the demand for practical use, since the target bacteria in a real food sample for routine screening are often of very low concentration, and the complex operations generally require well-trained technicians and professional laboratories. Various attempts have been made on continuous-flow magnetic separation to achieve effective and automatic extraction of DNA from large volume [27,28,29]. A typical example is reported by Kwon et al. [30] using magnetic particles to extract the target DNA from a bacterial sample with a high efficiency of 90%. However, the MSBs were aggregated at the external magnetic field, and only the MSBs at the surface still retained the ability to capture the DNA. Thus, a rotating magnetic separation field was used to form MSB chains for continuous-flow capture of the DNA, resulting in an extraction efficiency that was on average 47% more than the conventional MSB-based extraction method [31]. An interesting study on microfluidic magnetic fluidized bed was conducted to enable dynamic, efficient, and simplified magnetic bead actuation for DNA extraction in a continuous-flow regime [32]. However, the low flow rate resulted in a long extraction time.

**Figure 1:**
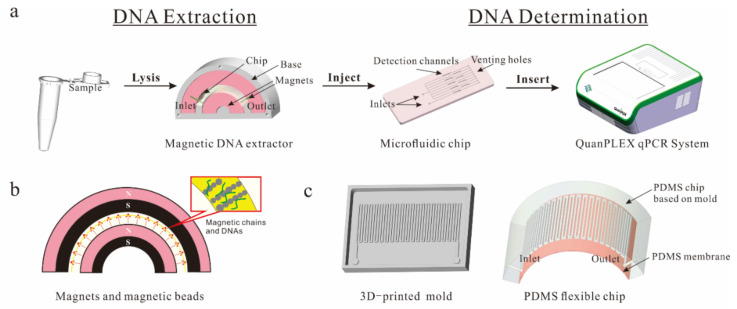
(**a**) The schematic for extraction and detection of the target nucleic acids; (**b**) The design of the concentric half-ring magnets; (**c**) The design of the 3D-printed mold and the flexible chip with the serpentine channel.

In our previous study, we have developed a coaxial channel for continuous-flow extraction of DNA from a large volume [13]. However, a large amount of MSBs were aggregated in the channel due to the strong and gradient magnetic field. In this study, we developed a new continuous-flow DNA extraction method and combined it with the microfluidic qPCR method for the rapid detection of *Salmonella*. As shown in Figure 1a, a magnetic DNA extractor was developed using two concentric half-ring magnets to first generate a homogeneous magnetic field and then form MSB chains in a serpentine channel of a flexible chip. Note that this magnetic field is highly homogeneous to ensure most positions can provide the magnetic chains. After the bacterial sample was lysed with protease K and injected into the channel, the DNA released from the bacterial cells were captured onto the MSB chains through electrostatic adsorption, followed by washing with ethanol, drying by air flow, and eluting in a small volume of deionized water to obtain purified and concentrated DNA templates. Finally, the DNA templates were injected into a microfluidic chip with lyophilized PCR reagents and determined using a commercial qPCR system.

## 2. Materials and Methods 

### 2.1. Materials

The ultrapure deionized water (18.2 MΩ·cm) was produced from Millipore Advantage A10 (Billerica, MA, USA. Phosphate-buffered saline solution (P5493, PBS, 10 times concentrated, diluted 10:1 by deionized water) was purchased from Sigma Aldrich (St. Louis, MO, USA). Bovine serum albumin (BSA) for blocking the channel was purchased from EM Science (Gibbstown, NJ, USA). Absolute ethanol for washing was purchased from Sinopharm (Beijing, China). The magnetic silica beads with a diameter of ≈1 μm for capturing the DNA were purchased from Huier Nano (HRXJ-0030, Zhengzhou, China). The nucleic acid detection kit, including reaction mixes, Taqman probes, and primers, for *Escherichia* coli (BD-1-210-1), *Vibrio* parahaemolyticus (BD-1-205), *Listeria* monocytogenes (BD-1-303), *Salmonella* typhimurium (BD-1-201-1), *Staphylococcus* aureus (BD-1-105), *Campylobacter* jejuni (BD-1-204-1), and *Bacillus* cereus (BD-1-420) were purchased from Huirui Biotech (Shanghai, China). The polydimethylsiloxane (PDMS) for fabricating the extraction chip was purchased from Dow Corning (Sylgard 184, Auburn, MI, USA). The PDMS membrane for bonding was purchased from Rogers (HT-6240, Carol Stream, IL, USA). The Objet24 3D printer and the material (VeroWhite plus RGD835) for printing the mold of the extraction chip were purchased from Stratasys (Eden Prairie, MN, USA).

### 2.2. Preparation of Bacterial Culture

The target bacteria, *Salmonella* typhimurium (ATCC14028), and other non-target bacteria were stored at −20 °C with 15% glycerol prior to use. They were cultured at 37 °C at 180 rpm for 12–16 h. Each bacterial culture was diluted with PBS to obtain the bacteria with different concentrations from 10^1^ to 10^8^ CFU/mL. To enumerate the viable bacteria, 100 µL of each dilution was plated onto the surface of Luria–Bertani agar plates. After incubation at 37 °C for 22–24 h, the number of bacterial colonies was counted, and the concentrations of bacteria were determined in the terms of colony-forming unit per milliliter (CFU/mL).

### 2.3. Fabrication of the Magnetic DNA Extractor

The magnetic DNA extractor plays the most important role in this method. As shown in Figure 1b, it consists of four parts: a magnetic field generator, a flexible extraction chip, a 3D-printed holder, and a peristaltic pump. The magnetic field was generated by two attractive concentric half-ring magnets (Outside magnet: outer diameter of 30 mm, inner diameter of 20 mm and depth of 5 mm; Inside magnet: outer diameter of 15 mm, inner diameter of 5 mm and depth of 5 mm; Material: Neodymium Iron Boron; Grade: N52). As shown in Figure 1b, the direction of the magnetic field is from the outer wall of the inside magnet to the inner wall of the outside magnet. When the MSBs are injected into the channel, they move along the magnetic field lines and form into chains. As shown in Figure 1c, the extraction chip is fabricated using the Object24 3D printer with the accuracy of 100 μm on both the X-axis and Y-axis and 28 μm on the Z-axis. The rectangle mold is 50 mm in length, 30 mm in width, and 5 mm in height with a serpentine channel, which is 10 mm in length, 0.5 mm in width, and 0.5 mm in height. The distance between the neighboring channels is 0.4 mm. The PDMS prepolymer and curing agent were first mixed at a ratio of 10:1 for 30 min and degassed for 20 min in vacuum. After that, the mixture was poured into the 3D-printed mold and cured at 65 °C overnight. Then, two holes with a diameter of 2 mm at the inlet and outlet were punched through the chip, respectively, after the solid PDMS was peeled out from the mold. Finally, the flexible PDMS chip was fabricated by bonding the PDMS channel to a PDMS membrane through surface plasma treatment (Harrick Plasma, Ithaca, NY, USA). The holder was fabricated by the 3D printer to house the half-ring magnets and the flexible chip. After the magnets were inserted into the holder, the flexible extraction chip was bent along the serpentine channel to match the arc cartridge between these two magnets. The peristaltic pump (BT100-2J&DG2B, Longer Pump, Baoding, China) was connected with the flexible chip to inject the solutions. 

### 2.4. Development of the Multiplex Microfluidic PCR Chip Preloaded with Lyophilized PCR Reagents

As shown in Figure 1a, the microfluidic PCR chip with a length of 79 mm, a width of 29 mm, and a height of 2.5 mm consists of 8 zones (6.5 mm × 1.5 mm × 0.2 mm for the top layer and 4.5 mm × 1.5 mm × 1 mm for the bottom layer with a space of 4.25 mm): 7 parallel detection zones from one inlet and 1 control zone from the other inlet. The channels are 0.2 mm in height and 0.2 mm in width. The inlets and venting holes are both 1 mm in diameter. 

The lyophilized PCR reagent included 3.1 µL of PCR probes and primers, 5.1 µL of PCR Master Mixes for amplification, 0.5 µL of trehalose, and 1.3 µL of 30% glucan 40 for reagent protection. The trehalose and glucan 40 were first prepared at 99 °C before injecting the lyophilized reagent into each channel carefully. After all the reagents were mixed, the surface of the microfluidic PCR chip was washed by absolute ethanol three times. Then, the microfluidic PCR chip was stored in the ultra-low temperature refrigerator for 6 h. Finally, the surface of the PCR chip was pasted with an anti-reflection glass by a double-coated tissue tape (9448A, 3M, Alexandria, MN, USA).

### 2.5. Extraction and Determination of Bacterial DNA

Prior to extraction, 200 µL of 1% BSA was injected into the extraction chip and kept for 30 min to block the channel, and 1 mL of PBS was used to wash the channel. Then, 20 µL of MSBs were first injected into the extraction chip and then inserted into the cartridge quickly. Due to the presence of the homogenous magnetic field, the MSBs were finally formed into MSB chains and ready to extract the DNA.

For DNA extraction, 1 mL of bacterial sample was first lysed with the lysis buffer including 300 µL of guanidine salt and surfactant, 10 µL of acryl carrier, and 5 µL of protease K, at 70 °C for 10 min. After the lysed bacterial sample was mixed with 300 µL of isopropanol, it was injected into the channel continuously, resulting in the capture of DNA onto the MSB chains. Then, 2 mL of absolute ethanol was injected into the channel for 4 min to wash the MSBs and remove the residual proteins, and the air was continuously injected into the channel for 30 min to remove the absolute ethanol. Finally, 100 µL of deionized water was injected to elute the DNA. To check the DNA quantity, the purity and concentration of the extracted DNA templates were measured by NanoDrop 2000 Spectrophotometer (Thermo Fisher, Waltham, MA, USA). For the manual method based on the instructions of the commercial reagent, the bacterial sample was first lysed for 10 min with the sample reagent of the proposed method and mixed with 300 µL of isopropanol before adding 20 µL of MSBs. After incubating for 10 min, the MSBs were separated by a magnetic extractor for 3 min and repeated 3 times. Then, the MSBs were washed by alcohol for 10 min and dried in the tube for another 15 min. Finally, the MSBs were eluted by 200 µL of deionized water for 10 min and separated for 3 min by the magnetic extractor to elute the DNA. 

For DNA determination, 84 µL of the extracted DNA was simultaneously injected into the 7 detection channels of the microfluidic PCR chip, i.e., each detection channel contained 12 µL of DNA, and 12 µL of PBS was injected into the other detection channel as a negative control. After a glass cover was used to seal the PCR chip to avoid aerosol pollution, it was finally placed into the qPCR system for DNA determination. The PCR reaction conditions were optimized by preprocessing at 50 °C for 2 min with uracil-N-glycosylase, predenaturation at 95 °C for 3 min, followed by 40 cycles of 95 °C for 5 s and 55 °C for 60 s. Three cycle threshold (Ct) values of parallel PCR tests were obtained and averaged for the determination of the original copies of the target DNA. The extracted DNA was also determined using the commercial StepOne qPCR system (Thermo Fisher, Waltham, MA, USA). In this system, 5 µL of extracted DNA, 12.5 µL of qPCR Master Mixes, and 7.5 µL of probes and primers were added into the PCR tube for DNA determination.

### 2.6. Detection of Salmonella Typhimurium in the Spiked Milk Samples

The pasteurized milk from a local supermarket was diluted 10 times before use, which was suggested by China’s food safety national standards. To prepare the spiked milk samples with different concentrations of *Salmonella* typhimurium, 100 µL of *Salmonella* typhimurium with a concentration from 10^1^ to 10^8^ CFU/mL was mixed with 900 µL of the prepared milk. Then, each spiked milk sample was lysed at 70 °C for 10 min and mixed with 300 µL of isopropanol, followed by DNA extraction using the magnetic DNA extractor and DNA determination using the microfluidic PCR chip.

## 3. Results and Discussions

### 3.1. Simulation of the Homogenous Magnetic Field

**Figure 2:**
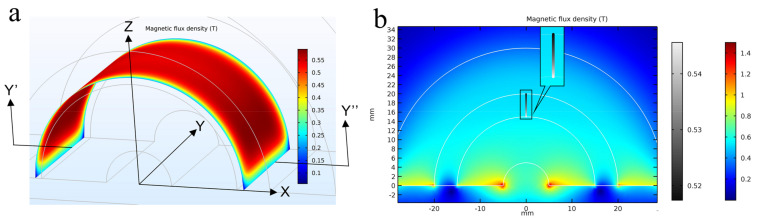
(**a**) The distribution of the magnetic field between these two half-ring magnets; (**b**) The cross-section distribution of the magnetic flux density in the center.

The distribution of the homogenous magnetic field in the serpentine channel is the key to forming the MSB chains and thus has a great impact on the DNA extraction efficiency. The 3D structure of the magnets was first drawn using SolidWorks (Waltham, MA, USA) and imported into COMSOL Multiphysics (Burlington, MA, USA) for simulation of the magnetic field. As shown in Figure 2a, the magnetic flux density at the center of these two half-ring magnets is almost homogeneous except for the edge of the magnets. To further understand the distribution of the magnetic field, the cross-section of the magnetic field at the center (Y’-Y’’) is simulated. As shown in Figure 2b, the magnetic flux density varies from 0.51 T to 0.54 T, indicating there is a homogenous magnetic field between these two magnets, and this allows the MSBs to form into MSB chains and thus prevent the aggregation of the MSBs, resulting in the increased opportunity for the MSBs to capture the target DNA.

### 3.2. Forming of the Magnetic Silica Bead Chains

**Figure 3:**
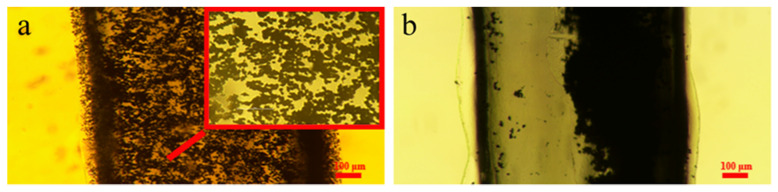
(**a**) The magnetic silica beads (MSB) chains in the serpentine channel under the homogeneous magnetic field; (**b**) The aggregated MSBs in the serpentine channel under the inhomogeneous magnetic field.

To verify the forming of MSB chains in the channel, a new method was developed to observe the MSB chains, because the chains could not be directly observed due to visual obstruction. First, the flexible extraction chip was inserted into the cartridge quickly, after the MSBs were injected into the serpentine channel, and the background solution was thoroughly drained. Then, the liquid agar, which was heated in a water bath at 90 °C for 20 min, was injected to fill in the channel and remained at room temperature for 24 h for solidification. Finally, the chip was cut in the middle along the long axis, while the image was taken from the side of the microchannel using an inverted fluorescence microscope (Nikon Ti-E, Tokyo, Japan). As shown in Figure 3a, the MSB chains were observed to distribute in the whole serpentine channel using the microscope, and the length of the MSB chains varies from 20 to 100 μm, which is consistent with the simulation. To further verify that this forming of the MSB chain is due to this homogenous magnetic field, an inhomogeneous magnetic field using a rectangle magnet (size: 20 mm × 20 mm × 5 mm, material: Neodymium Iron Boron, grade: N52) was used to capture the MSBs for comparison. As shown in Figure 3b, the MSBs were aggregated against one wall of this serpentine channel, making the partially buried MSBs unable to capture the flowing DNA and thus lower the capture efficiency of the target DNA. 

### 3.3. Optimization of the DNA Extraction

Prior to optimization, different concentrations (C) of *Salmonella* typhimurium from 10^3^ CFU/mL to 10^8^ CFU/mL were prepared for DNA extraction using the conventional magnetic silica bead separation and DNA detection using the commercial StepOne quantitative PCR instrument to determine the unknown DNA concentration. The cycle threshold values (Ct) for different concentrations were obtained and related with the bacterial concertation as Ct = −3.71 × log (C) + 48.62 (R^2^ = 0.997). Moreover, the efficiency (E) for DNA extraction was defined as E = Ce/Co × 100%, where Ce and Co are the concentrations of the extracted DNA and original DNA and used to evaluate the magnetic DNA extractor. 

**Figure 4:**
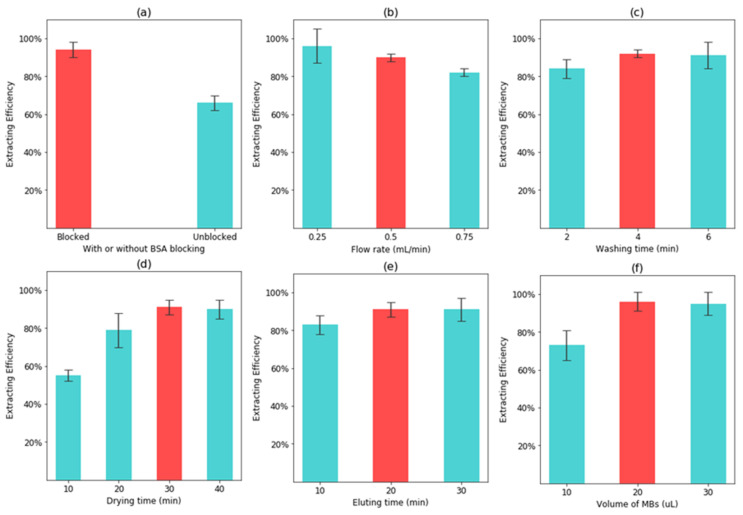
Optimization of the DNA extraction. (**a**) Extraction efficiency with and without bovine serum albumin (BSA) blocking (n = 3); (**b**) Extraction efficiency at different flow rates (n = 3); (**c**) Extraction efficiency at different washing times (n = 3); (**d**) Extraction efficiency at different drying times (n = 3); (**e**) Extraction efficiency at different eluting times (n = 3); (**f**) Extraction efficiency at different volumes of MSBs (n = 3).

Nonspecific adsorption of nucleic acid often occurs in PDMS channels [33]. Thus, 200 µL of 1% BSA was used to block the PDMS channel. To verify the blocking effect, the lysed DNA templates (concentration: 10^6^ CFU/mL) were injected into the channel with and without BSA blocking, respectively, which was followed by detection using the StepOne PCR. The original DNA was also detected for comparison. As shown in Figure 4a, only 6.4% of the total DNA templates are lost in the channel with the BSA block, while 34% of those are lost in the channel without the BSA block. This indicates the BSA block is effective to reduce the nonspecific adsorption of DNA to the PDMS channel.

The flow rate has a great impact on the DNA extraction. Thus, different flow rates from 0.25 to 0.75 mL/min were compared to extract the nucleic acids of target bacteria at the concentration of 10^5^ CFU/mL. After the same concentration of target bacteria was lysed to release the DNA, the lysed bacterial sample was injected into the channel at different flow rates and captured onto the MSB chains, which was followed by elution for DNA determination using qPCR. As shown in Figure 4b, when the flow rate decreases from 0.75 to 0.5 mL/min, the DNA extraction efficiency increases from 81% to 91%, because the MSBs have more chance to capture the DNA at a lower flow rate. However, when the flow rate decreases to 0.25 mL/min, only a slight increase on the extraction efficiency (94%) is obtained. Thus, the optimal flow rate of 0.25 mL/min was used in this study.

Washing, drying, and eluting are also important to the DNA extraction. Thus, different washing times (from 2 to 6 min), drying times (from 10 to 40 min), and eluting times (from 10 to 30 min) were optimized at the optimal flow rate of 0.5 mL/min. As shown in Figure 4c, the extraction efficiency increases from 83.6% to 91.9% when the washing time changes from 2 to 4 min. No obvious increase on the extraction efficiency is found when the washing time increases to 6 min. Therefore, the optimal washing time of 4 min was used to remove the impurities. As shown in Figure 4d, the extraction efficiency increases from 55% to 90% when the drying time changes from 10 to 30 min. This is because some ethanol is still present in the DNA elution solution at short drying time, resulting in partial inhibition of the PCR reaction. However, the extraction efficiency remains at the same level (91%) when the drying time increases to 40 min. Thus, the optimal drying time of 30 min was used in this study. As shown in Figure 4e, the extraction efficiency increases from 82% to 91% when the elution time changes from 10 min to 20 min. However, the extraction efficiency does not increase when the elution time increases to 30 min, indicating that almost all the DNA has been eluted. Thus, the optimal eluting time of 20 min was used in this study. The amount of MSBs plays a key role in the DNA extraction. Thus, different volumes of MSBs (concentration: 100 mg/mL) from 10 to 30 µL were compared to extract the DNA. As shown in Figure 4f, the extraction efficiency increases from 72% to 95%, when the volume changes from 10 to 20 µL. However, no increase on the extraction efficiency is obtained when the volume increases to 30 µL, indicating that 20 µL is sufficient to extract almost all the DNA. Thus, the optimal volume of 20 µL was used in this study. At the optimal conditions, more than 90% of the DNA could be extracted from the bacterial sample within 65 min, including lysing for 10 min, adsorption for 1 min, washing for 4 min, drying for 30 min, and eluting for 20 min. In addition, the extracted DNA optimal density (OD) 260/280 ratio measured by a spectrophotometer was 1.94, which indicates that the extracted DNA was pure.

Compared with the manual separation method in a large volume of sample, the continuous-flow DNA extraction does not show obvious advantages on the processing time if we use a larger tube and strong magnetic separator in the manual separation. However, it has three beneficial aspects. First, it uses fewer MSBs because the incubation space is constant. Secondly, this procedure is simpler due to the use of a half-automatic pump. Thirdly, it can process the sample with various volumes; however, the conventional magnetic separator can only extract the magnetic target from a constant volume of sample.

### 3.4. Detection of Salmonella Typhimurium

**Figure 5:**
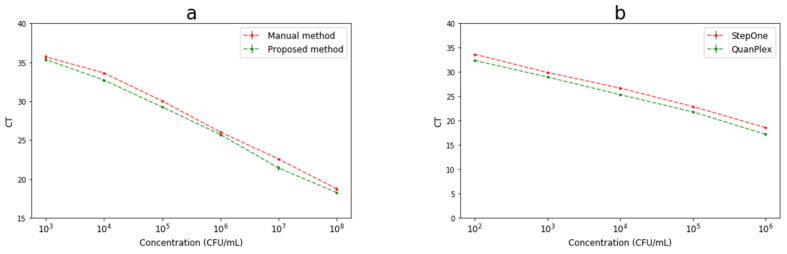

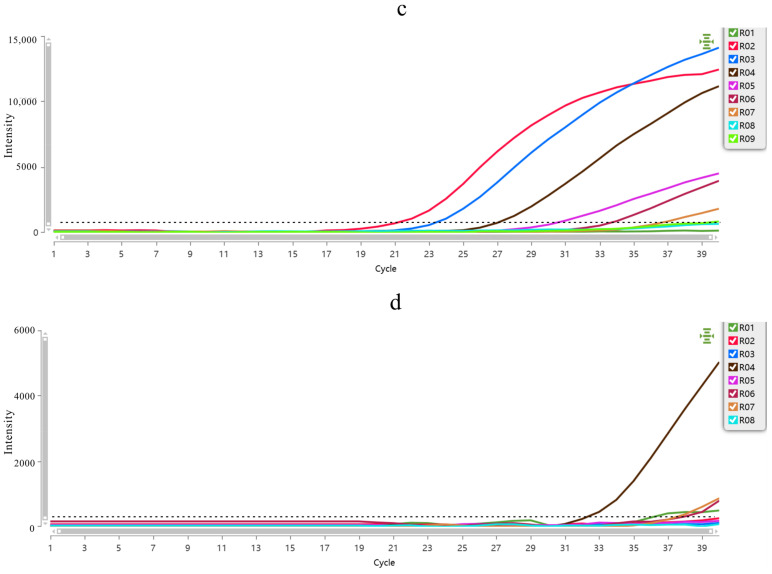
Detection of *Salmonella* using this magnetic extractor and microfluidic PCR chip. (**a**) Comparison of this magnetic extractor with the manual magnetic separation method; (**b**) Comparison of this microfluidic PCR chip with the commercial StepOne qPCR instrument; (**c**) Detection of different concentrations of *Salmonella* in milk using the magnetic extractor and microfluidic PCR chip. (R01 stands for control and R02 to R09 stand for 1 × 10^8^ CFU/mL to 1 × 10^1^ CFU/mL, respectively); (**d**) Detection of different bacteria using this magnetic extractor and microfluidic PCR chip (R01 to R08 stand for *Escherichia* coli, *Vibrio* parahaemolyticus, *Listeria* monocytogenes, *Salmonella* Typhimurium, *Staphylococcus* aureus, *Campylobacter* jejuni, *Bacillus* cereus, and blank control, respectively).

To verify the feasibility of this proposed DNA extraction and detection method, different concentrations of *Salmonella* Typhimurium from 1 × 10^3^ CFU/mL to 1 × 10^8^ CFU/mL were extracted using both this proposed magnetic extractor and the manual methods, and the extracted DNA was detected using both this microfluidic PCR chip and the commercial StepOne qPCR instrument. As shown in Figure 5a, the Ct values for these two DNA extraction methods at different concentrations are very close, indicating that these two methods have a good consistence, and thus, this magnetic extractor is suitable for DNA extraction. As shown in Figure 5b, although the Ct value for the proposed microfluidic PCR chip at each concentration is basically ≈0.65 lower than that for the commercial qPCR instrument, the Ct values for both qPCR methods have a good linear relationship with the logarithm of the bacterial concentration, indicating that this proposed microfluidic PCR chip is suitable for DNA detection. Moreover, the proposed microfluidic PCR system requires less time of ≈48 min compared to the StepOne qPCR instrument of ≈55 min with the same settings because of the faster heating rate. Another advantage was that the microfluidic PCR system offers a personalized design for detection. Thus, the calibration model of this proposed method combining this magnetic DNA extractor and microfluidic PCR chip can be expressed as Ct = −1.63 × log (C) + 40.163 (R^2^ = 0.997). According to three times of signal-to-noise ratio, this lower detection limit was calculated as 10^2^ CFU/mL. The high sensitivity mainly contributed to three aspects: (1) the homogeneous magnetic field was generated using concentric half-ring magnets, resulting in the increase on the opportunity for the MSBs to capture the DNA; (2) the large volume (10 mL) of the bacterial sample was used, resulting in the extraction of more target DNA; and (3) the microfluidic PCR chip was developed for automatic operation in PCR reaction, resulting in the improvement of the signal-to-noise ratio.

To evaluate the applicability of this proposed method for the detection of *Salmonella* Typhimurium in food samples, different concentrations of *Salmonella* Typhimurium were added into milk to prepare the spiked bacterial samples, 10 mL of which were first extracted using this magnetic DNA extractor; then, they were eluted in 100 µL of diluted water and finally detected using this microfluidic PCR chip. As shown in Figure 5c, the Ct values for different concentrations of *Salmonella* Typhimurium from 10^3^ to 10^8^ CFU/mL were 35.91, 33.08, 29.72, 26.51, 23.15, and 20.92, respectively. To evaluate the specificity of this proposed method, six non-target bacteria, including *Escherichia* coli O157:H7, *Vibrio* parahaemolyticus, *Listeria* monocytogenes, *Staphylococcus* aureus, *Campylobacter* jejuni, and *Bacillus* cereus, and the target bacteria, *Salmonella* Typhimurium, at the same concentration of 10^6^ CFU/mL were detected using this proposed method. As shown in Figure 5d, only the target bacteria has an effective Ct value, and all six non-target bacteria do not have Ct values, indicating that this method has good specificity.

## 4. Conclusions

In this study, a new magnetic extractor and microfluidic PCR were successfully developed using 3D printing and magnetic silica beads. They have been demonstrated with the ability to extract more than 90% of the target DNA from a large volume (10 mL) of bacterial sample and detect the bacteria as low as 10^2^ CFU/mL. The homogeneous magnetic field generated by two concentric semicircle magnets was able to form the MSBs into chains and thus enhance the capture of flowing DNA. The microfluidic PCR chip with lyophilized amplification reagents was consistent with the commercial qPCR instrument, and it has the potential to provide rapid, sensitive, and simultaneous detection of multiple foodborne pathogens.

## Data Availability

Not applicable.
